# MicroRNA 33 Regulates the Population of Peripheral Inflammatory Ly6C^high^ Monocytes through Dual Pathways

**DOI:** 10.1128/MCB.00604-17

**Published:** 2018-06-28

**Authors:** Osamu Baba, Takahiro Horie, Tetsushi Nakao, Daihiko Hakuno, Yasuhiro Nakashima, Hitoo Nishi, Yasuhide Kuwabara, Masataka Nishiga, Tomohiro Nishino, Yuya Ide, Fumiko Nakazeki, Satoshi Koyama, Masahiro Kimura, Ritsuko Hanada, Masahiro Kawahara, Takeshi Kimura, Koh Ono

**Affiliations:** a Department of Cardiovascular Medicine, Graduate School of Medicine, Kyoto University, Kyoto, Japan; b Department of Hematology and Oncology, Graduate School of Medicine, Kyoto University, Kyoto, Japan

**Keywords:** HDL-C, atherosclerosis, microRNA, monocytes, stem cells

## Abstract

MicroRNA 33 (miR-33) targets ATP-binding cassette transporter A1 (ABCA1), and its deficiency increases serum high-density lipoprotein (HDL)-cholesterol (HDL-C) and ameliorates atherosclerosis. Although we previously reported that miR-33 deficiency increased peripheral Ly6C^high^ monocytes on an ApoE-deficient background, the effect of miR-33 on the monocyte population has not been fully elucidated, especially in a wild-type (WT) background. We found that Ly6C^high^ monocytes in miR-33^−/−^ mice were decreased in peripheral blood and increased in bone marrow (BM). Expansion of myeloid progenitors and decreased apoptosis in Lin^−^ Sca1^+^ c-Kit^+^ (LSK) cells were observed in miR-33^−/−^ mice. A BM transplantation study and competitive repopulation assay revealed that hematopoietic miR-33 deficiency caused myeloid expansion and increased peripheral Ly6C^high^ monocytes and that nonhematopoietic miR-33 deficiency caused reduced peripheral Ly6C^high^ monocytes. Expression of high-mobility group AT-hook 2 (HMGA2) targeted by miR-33 increased in miR-33-deficient LSK cells, and its knockdown abolished the reduction of apoptosis. Transduction of human apolipoprotein A1 and ABCA1 in WT mouse liver increased HDL-C and reduced peripheral Ly6C^high^ monocytes. These data indicate that miR-33 deficiency affects distribution of inflammatory monocytes through dual pathways. One pathway involves the enhancement of *Hmga2* expression in hematopoietic stem cells to increase Ly6C^high^ monocytes, and the other involves the elevation of HDL-C to decrease peripheral Ly6C^high^ monocytes.

## INTRODUCTION

MicroRNAs (miRs) are small nonprotein coding RNAs that bind to specific mRNAs and inhibit their translation or promote their degradation (1–3). We along with other groups reported that miR-33 is encoded in an intron of sterol regulatory element-binding protein 2 (SREBP2) and targets ATP-binding cassette transporter A1 (ABCA1), which facilitates the efflux of cellular cholesterol to apolipoprotein A1 (ApoA1) to form pre-β-high-density lipoprotein cholesterol (HDL-C) (4–7). In fact, miR-33-deficient (miR-33^−/−^) mice showed an increase in both ABCA1 expression and HDL-C level. We also confirmed that atherosclerotic plaque formation was ameliorated in miR-33^−/−^ *Apoe*^−/−^ mice compared with that in *Apoe*^−/−^ mice (8).

On the other hand, monocytes play an important role in atherosclerotic plaque progression (9), and there are at least two monocyte subsets in both humans and mice. One subset is comprised of classical inflammatory monocytes, which are typically identified as Ly6C^high^ monocytes in mice and as CD14^+^ CD16^−^ monocytes in humans. The other subset is comprised of nonclassical monocytes, which are identified as Ly6C^low^ monocytes in mice and as CD14^low^ CD16^+^ monocytes in humans (10). There are genetic similarities between these human and mouse monocyte subsets (11, 12). Furthermore, Ly6C^high^ monocytes are mainly recruited into atherosclerotic plaques in *Apoe*^−/−^ mice on a high-fat diet (13, 14). Meanwhile, there is a report that the percentage and number of classical CD14^+^ CD16^−^ monocytes are positively correlated with the incidence of cardiovascular events (15).

In a previous study, we found that the proportion of Ly6C^high^ monocytes in peripheral blood (PB) was increased in miR-33^−/−^ *Apoe*^−/−^ mice compared with the level in *Apoe*^−/−^ mice (8). However, the effect of miR-33 on leukocyte population and bone marrow (BM) function is still unclear, especially under ApoE-sufficient conditions. Moreover, although atherosclerotic plaque progression was suppressed in miR-33^−/−^ *Apoe*^−/−^ mice, the increase in Ly6C^high^ monocyte proportion might have an adverse effect on atherosclerotic plaque formation. Previous studies that tried to repress atherosclerotic plaque progression with anti-miR-33 oligonucleotides have shown inconsistent results (16–19). Thus, we speculated that the effect of miR-33 on monocyte subsets might offer a clue to elucidate this inconsistency. Therefore, we analyzed the effect of miR-33 on monocyte distribution and BM function, including hematopoiesis.

In the present study, we found that miR-33 regulates the population of peripheral inflammatory Ly6C^high^ monocytes through both hematopoietic and nonhematopoietic pathways. This is a novel report showing that one microRNA regulates one phenotype through two different pathways which have opposite effects. The miR-33-deficient hematopoietic pathway increases peripheral Ly6C^high^ monocytes through the expansion of myeloid progenitors, including hematopoietic stem cells. In contrast, the miR-33-deficient nonhematopoietic pathway decreases myeloid progenitors by suppressing their emigration from BM. The balance of these two components could determine the number and proportion of peripheral Ly6C^high^ monocytes under an miR-33-deficient condition and may affect inflammatory responses such as atherosclerosis formation.

## RESULTS

### miR-33 deficiency reduced peripheral Ly6C^high^ monocytes and increased Ly6C^high^ monocytes in BM under ApoE-sufficient conditions.

**FIG 1:**
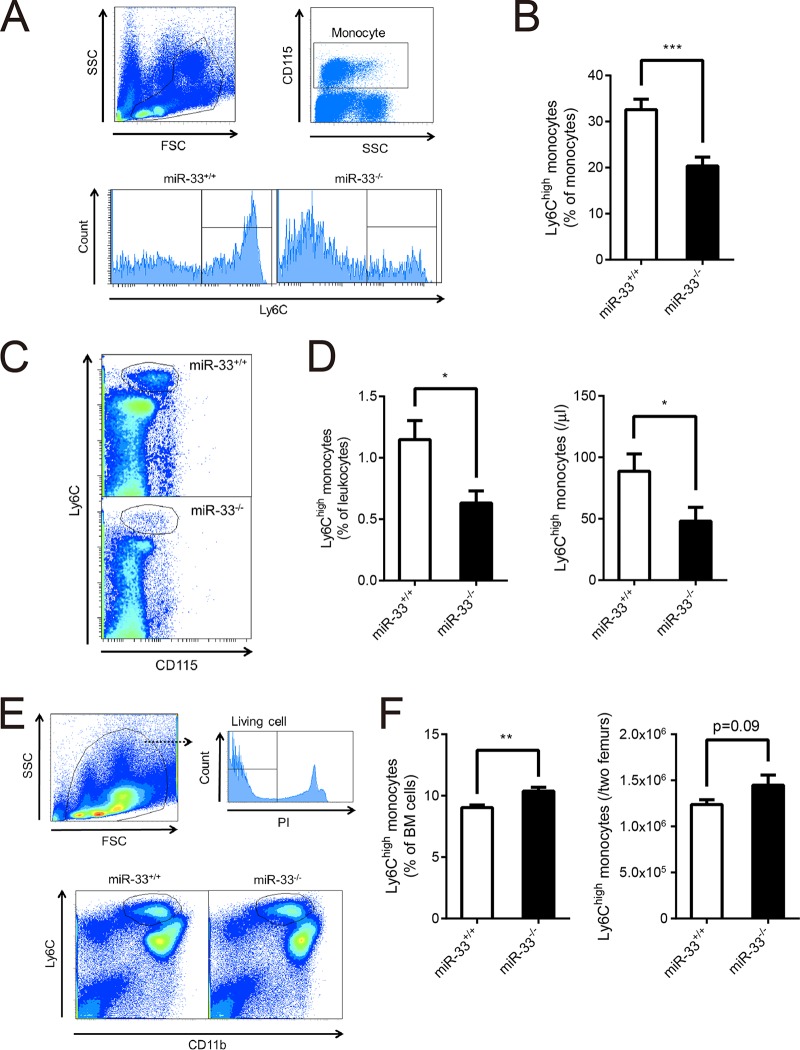
Ly6C^high^ monocytes in PB were decreased, and those in BM were increased in miR-33^−/−^ mice. (A) Representative histograms of the proportion of Ly6C^high^ monocytes in peripheral monocytes from miR-33^+/+^ and miR-33^−/−^ mice. (B) The proportion of Ly6C^high^ monocytes in peripheral monocytes from miR-33^+/+^ and miR-33^−/−^ mice (miR-33^+/+^, *n* = 15 mice; miR-33^−/−^, *n* = 14 mice). (C) Representative dot plots of Ly6C^high^ monocytes in PB from miR-33^+/+^ and miR-33^−/−^ mice. (D) The proportion (left) and the number (right) of Ly6C^high^ monocytes in PB from miR-33^+/+^ and miR-33^−/−^ mice (miR-33^+/+^, *n* = 15 mice; miR-33^−/−^, *n* = 14 mice). (E) The scheme for gating of Ly6C^high^ monocytes in BM and representative dot plots of Ly6C^high^ monocytes in BM. (F) The proportion (left) and the number (right) of Ly6C^high^ monocytes in BM from miR-33^+/+^ and miR-33^−/−^ mice (*n* = 15 per group). All data are shown as means ± SEM. *, *P* < 0.05; **, *P* < 0.01, ***, *P* < 0.001 (by Student's *t* test). SSC, side scatter; FSC, forward scatter.

At first, we assessed PB cell counts of miR-33^−/−^ mice and their littermates (miR-33^+/+^ mice) at 8 weeks of age (Table 1). Leukocyte counts were almost the same between these two groups of mice. Mean corpuscular volume (MCV) and mean corpuscular hemoglobin (MCH) of miR-33^−/−^ mice were significantly higher than those of miR-33^+/+^ mice although the reason for this difference is still unclear. We analyzed the population of peripheral leukocytes by flow cytometry. Monocytes and granulocytes tended to decrease in miR-33^−/−^ mice (Table 2). Next, we assessed the proportion of Ly6C^high^ monocytes. Strikingly, this proportion was significantly reduced in miR-33^−/−^ mice compared to the level in miR-33^+/+^ mice, in contrast to a previous result where the level was significantly increased in miR-33^−/−^ *Apoe*^−/−^ mice compared to the level in miR-33^+/+^ *Apoe*^−/−^ mice (Fig. 1A and B) (8). The percentage and absolute number of Ly6C^high^ monocytes in PB also decreased in miR-33^−/−^ mice (Fig. 1C and D). To assess whether the reduced production in BM or repressed emigration from BM caused this decrease in peripheral Ly6C^high^ monocytes in miR-33^−/−^ mice, we analyzed the population of Ly6C^high^ monocytes in BM. Results showed that Ly6C^high^ monocytes were increased in miR-33^−/−^ mice compared to the level in miR-33^+/+^ mice (Fig. 1E and F). These data indicate that the egress of Ly6C^high^ monocytes from BM is suppressed in miR-33^−/−^ mice.

**TABLE 1:** Peripheral blood cell counts of miR-33^+/+^ and miR-33^−/−^ mice

Parameter^*a*^	Value for the parameter by genotype (mean ± SD)		*P*^*b*^
	miR-33^+/+^ (*n* = 19)	miR-33^−/−^ (*n* = 19)	
No. of leukocytes (10^9^/liter)	5.60 ± 1.53	5.54 ± 1.20	0.90
No. of RBC (10^12^/liter)	9.50 ± 0.69	9.13 ± 0.52	0.073
Hemoglobin (g/dl)	12.9 ± 1.2	12.9 ± 1.2	0.86
Hematocrit (%)	49.5 ± 3.7	49.1 ± 2.5	0.70
No. of platelets (10^9^/liter)	912 ± 84	946 ± 91	0.25
MCV (fl)	52.1 ± 0.82	53.8 ± 0.45	<0.0001***
MCH (pg)	13.6 ± 0.56	14.07 ± 0.62	0.02*

a RBC, red blood cells; MCV, mean corpuscular volume; MCH, mean corpuscular hemoglobin.

b Significance was determined by Student's *t* test. *, *P* < 0.05; ***, *P* < 0.001.

**TABLE 2:** The number of each peripheral leukocyte population in miR-33^+/+^ and miR-33^−/−^ mice

Leukocyte population	No. of cells/μl by genotype^*a*^		*P*
	miR-33^+/+^	miR-33^−/−^	
Monocytes	244 ± 30	197 ± 29	0.26
Granulocytes	450 ± 96	342 ± 31	0.28
T cells	1,291 ± 92	1,257 ± 76	0.78
B cells	3,101 ± 188	3,083 ± 173	0.95

a Values are means ± SEM (*n* = 19 or 20 per group).

### miR-33 deficiency increased myeloid progenitors by suppressing apoptosis in hematopoietic stem cells.

**FIG 2:**
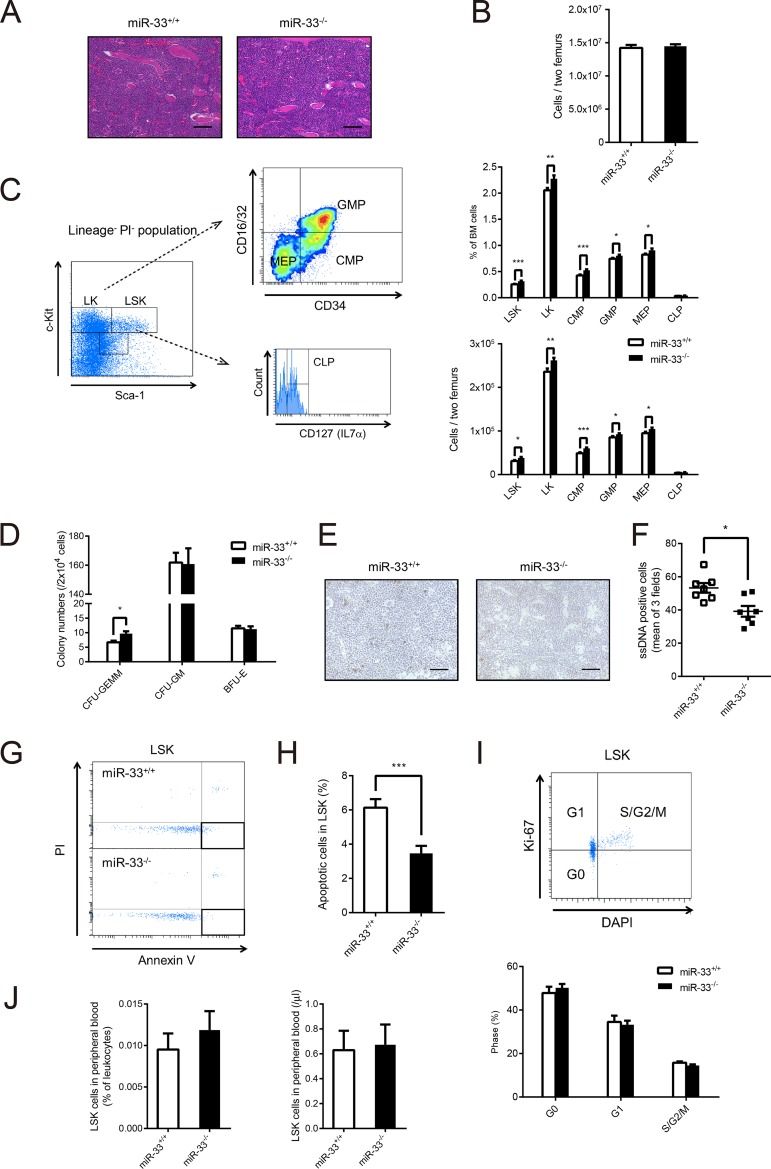
miR-33 deficiency reduced apoptosis in LSK cells and increased in LSK cells and committed progenitor cells in BM. (A) Hematoxylin and eosin staining of BM from miR-33^+/+^ and miR-33^−/−^ mice. Scale bars, 50 μm. (B) Total number of BM cells obtained from two femurs from miR-33^+/+^ and miR-33^−/−^ mice (*n* = 28 per group). (C) The scheme for gating of LSK and LK cells and myeloid committed progenitor cells in BM, and the proportion (upper) and the number (bottom) of each cell population in miR-33^+/+^ and miR-33^−/−^ mice (*n* = 18 to 28 per group). (D) The number of colonies, as indicated, grown from 2 × 10^4^ BM cells from miR-33^+/+^ and miR-33^−/−^ mice (miR-33^+/+^, *n* = 12; miR-33^−/−^, *n* = 13 mice). GEMM, granulocyte, erythroid, macrophage, megakaryocyte; GM, granulocyte, macrophage; BFU-E, burst-forming unit-erythroid. (E) Representative microscopic images of single-stranded DNA staining of BM from miR-33^+/+^ and miR-33^−/−^ mice. Scale bars, 50 μm. (F) The number of single-stranded DNA (ssDNA)-positive cells per field at a magnification of ×400 (each number is a mean of positive cells in three fields) (*n* = 7 per group). *, *P* < 0.05 (by Mann-Whitney *U* test). (G) Representative dot plots of apoptosis in LSK cells from miR-33^+/+^ and miR-33^−/−^ mice. (H) The proportion of apoptosis in LSK cells from miR-33^+/+^ and miR-33^−/−^ mice (*n* = 18 per group). (I) Representative dot plots of cell cycle analysis with DAPI and anti-Ki-67 antibody and the result of the cell cycle analysis of LSK cells from miR-33^+/+^ and miR-33^−/−^ mice (*n* = 11 per group). (J) The proportion (left) and the number (right) of LSK cells in PB from miR-33^+/+^ and miR-33^−/−^ mice (*n* = 10 per group). All data are shown as means ± SEM. *, *P* < 0.05; **, *P* < 0.01, ***, *P* < 0.001 (by Student's *t* test except for the data in panel F).

BM Ly6C^high^ monocytes were increased in miR-33^−/−^ mice. Therefore, we analyzed the population of hematopoietic progenitor cells in miR-33^−/−^ mice. At first, we assessed the cellularity of BM. There were no histological differences between the two groups of mice (Fig. 2A), and the numbers of BM cells counted by a cell counter were almost the same (Fig. 2B). Next, we analyzed the population of hematopoietic progenitors by flow cytometry (Fig. 2C). Consequently, the percentages and numbers of Lin^−^ Sca1^+^ c-Kit^+^ (LSK) cells, Lin^−^ Sca1^−^ c-Kit^+^ (LK) cells, common myeloid progenitors (CMPs), granulocyte-macrophage progenitors (GMPs), and megakaryocyte-erythroid progenitors (MEPs) were significantly increased in miR-33^−/−^ mice compared with the levels in miR-33^+/+^ mice. On the other hand, the population of common lymphoid progenitors (CLPs) did not change (Fig. 2C). Moreover, a colony-forming assay showed that the number of CFU-granulocyte, erythroid, macrophage, megakaryocyte (CFU-GEMM) colonies derived from miR-33^−/−^ BM cells was significantly increased compared to that from miR-33^+/+^ BM cells (Fig. 2D).

Next, we tried to reveal what caused this increase in LSK cells in BM of miR-33^−/−^ mice. The number of hematopoietic stem cells is mainly regulated by apoptosis, self-renewal, differentiation, and emigration (20). It had already been demonstrated that differentiated myeloid progenitors in BM were increased in miR-33^−/−^ mice (Fig. 2C). Thus, we assessed apoptosis, self-renewal, and emigration of LSK cells in miR-33^−/−^ mice. Apoptotic cells indicated by single-stranded DNA staining were significantly decreased in BM from miR-33^−/−^ mice (Fig. 2E and F). Then, we analyzed apoptotic cells in LSK cells by flow cytometry using annexin V and propidium iodide (PI). Results showed that apoptotic cells, represented as PI-negative and annexin V-positive cells, were significantly reduced in miR-33-deficient LSK cells (Fig. 2G and H). On the other hand, cell cycle analysis of LSK cells by flow cytometry using 4′,6′-diamidino-2-phenylindole (DAPI) and an anti-Ki-67 antibody did not show any differences between miR-33^−/−^ mice and their littermates (Fig. 2I). Although we analyzed the population of peripheral LSK cells to assess the emigration of LSK cells from the BM, there were also no differences between the two groups of mice (Fig. 2J). Taken together, these results show that miR-33 deficiency causes expansion of hematopoietic stem cells by suppressing their apoptosis and subsequently increases myeloid progenitors in BM.

### Hematopoietic factors in miR-33^−/−^ mice caused an increase in myeloid progenitors and Ly6C^high^ monocytes in BM, and nonhematopoietic factors in miR-33^−/−^ mice caused a decrease in peripheral Ly6C^high^ monocytes.

**FIG 3:**
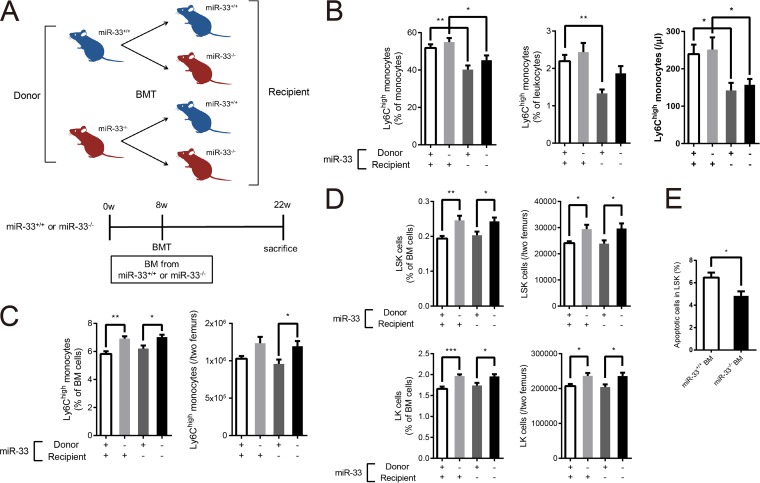
Hematopoietic miR-33 deficiency increased LSK, LK cells, and Ly6C^high^ monocytes in BM, and nonhematopoietic miR-33 deficiency reduced peripheral Ly6C^high^ monocytes. (A) Experimental protocol for BMT. w, weeks. (B) The proportion of Ly6C^high^ monocytes in peripheral monocytes (left) and the proportion (middle) and the number (right) of Ly6C^high^ monocytes in PB from four groups of mice (*n* = 12 to 19 per group). (C) The proportion (left) and the number (right) of Ly6C^high^ monocytes in BM from four groups of mice (*n* = 10 to 12 per group). (D) The proportion and the number of LSK (upper left and right) and LK (lower left and right) cells in BM from four groups of mice (*n* = 26 to 29 per group). (E) The proportion of apoptosis in LSK cells from miR-33^+/+^ recipient mice transplanted with miR-33^+/+^ or miR-33^−/−^ BM (miR-33^+/+^, *n* = 12 BM; miR-33^−/−^, *n* = 10 BM). All data are shown as means ± SEM. *, *P* < 0.05; **, *P* < 0.01, ***, *P* < 0.001 (by 1-way ANOVA with the Turkey *post hoc* test for panels A to D); *, *P* < 0.05 (by Student's *t* test for panel E).

To determine whether these phenotypes were mainly due to hematopoietic factors (donor's phenotype) or nonhematopoietic factors (recipient's phenotype) in miR-33^−/−^ mice, we generated four groups of mice using bone marrow transplantation (BMT) (Fig. 3A). BMT was performed in mice at 8 weeks of age, and then PB and BM were analyzed at 14 weeks post-BMT. Successful hematopoietic reconstitution after BMT was confirmed by PCR amplification of the BM and tail genomes (data not shown). In consequence, peripheral Ly6C^high^ monocytes were reduced in miR-33-deficient recipient mice regardless of the phenotype of the donors (Fig. 3B). Thus, the reduction of peripheral Ly6C^high^ monocytes in miR-33^−/−^ mice is caused by nonhematopoietic factors. On the other hand, Ly6C^high^ monocytes in BM were increased in mice transplanted with miR-33-deficient BM (Fig. 3C) regardless of the recipients' phenotypes. This indicates that the increase in Ly6C^high^ monocytes in BM is due to hematopoietic factors. Moreover, LSK and LK cells, which consist of CMPs, GMPs, and MEPs, were also increased in mice transplanted with miR-33-deficient BM (Fig. 3D). Thus, miR-33-deficient hematopoietic factors also cause the increase in LSK and LK cells. Additionally, apoptosis in LSK cells in miR-33^+/+^ mice transplanted with miR-33^−/−^ BM was significantly decreased compared with the level in miR-33^+/+^ mice transplanted with miR-33^+/+^ BM (Fig. 3E). Taken together, these results show that hematopoietic factors in miR-33^−/−^ mice suppress apoptosis in LSK cells and then induce myeloid expansion.

**FIG 4:**
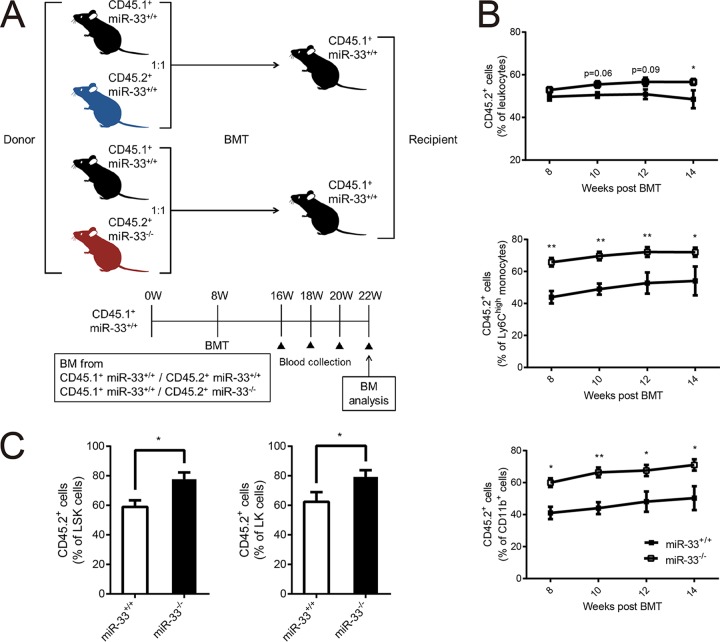
Hematopoietic miR-33 deficiency increases peripheral Ly6C^high^ monocytes without nonhematopoietic miR-33 deficiency. (A) Experimental protocol for the competitive repopulation assay. (B) The percentage of CD45.2^+^ leukocytes, Ly6C^high^ monocytes, and CD11b^+^ myeloid cells, as indicated, in PB from CD45.1^+^ miR-33^+/+^ mice transplanted with equally mixed CD45.1^+^ miR-33^+/+^ and CD45.2^+^ miR-33^+/+^ (miR-33^+/+^ group) or CD45.2^+^ miR-33^−/−^ BM (miR-33^−/−^ group) cells (miR-33^+/+^ group, *n* = 5; miR-33^−/−^ group, *n* = 14). (C) The percentage of CD45.2^+^ LSK and LK cells in the miR-33^+/+^ group and the miR-33^−/−^ group (miR-33^+/+^ group, *n* = 5;, miR-33^−/−^ group, *n* = 14). All data are shown as means ± SEM. *, *P* < 0.05; **, *P* < 0.01 (by Mann-Whitney *U* test).

A competitive repopulation assay was performed (Fig. 4A) to confirm these results. We mixed the BM cells obtained from CD45.1^+^ miR-33^+/+^ mice and CD45.2^+^ miR-33^+/+^ or CD45.2^+^ miR-33^−/−^ mice in a 1:1 ratio and transplanted them into CD45.1^+^ miR-33^+/+^ mice. In detail, we compared CD45.1^+^ miR-33^+/+^ mice transplanted with a mixture of CD45.1^+^ miR-33^+/+^ and CD45.2^+^ miR-33^+/+^ BM (miR-33^+/+^ group) and CD45.1^+^ miR-33^+/+^ mice transplanted with CD45.1^+^ miR-33^+/+^ and CD45.2^+^ miR-33^−/−^ BM (miR-33^−/−^ group). BMT was performed in mice at 8 weeks of age. The proportion of CD45.2^+^ cells in PB was assessed every 2 weeks from 8 to 14 weeks post-BMT, and then the proportion of CD45.2^+^ hematopoietic progenitor cells in BM was assessed at 14 weeks post-BMT. These results showed that the percentage of CD45.2^+^ leukocytes in PB was about 50% in the miR-33^+/+^ group, and this value tended to be higher in miR-33^−/−^ group. In particular, the proportions of CD 45.2^+^ Ly6C^high^ monocytes and CD11b^+^ myeloid cells in PB were significantly higher in the miR-33^−/−^ group than in the miR-33^+/+^ group (Fig. 4B). These results indicate that peripheral Ly6C^high^ monocytes in miR-33^−/−^ mice can increase without miR-33 deficiency in nonhematopoietic cells. Since most peripheral leukocytes are lymphocytes in mice and since their population did not change in miR-33^−/−^ mice (Table 2), the increase in the percentage of CD45.2^+^ cells in leukocytes might be less significant than that in Ly6C^high^ monocytes and CD11b^+^ myeloid cells in this experiment. Finally, the analysis of hematopoietic progenitor cells in BM showed that the percentage of CD 45.2^+^ LK and LSK cells was higher in the miR-33^−/−^ group, as expected (Fig. 4C). Taking these results together, miR-33 deficiency in hematopoietic stem cells increases peripheral Ly6C^high^ monocytes through myeloid expansion. Meanwhile, miR-33 deficiency in nonhematopoietic cells decreases Ly6C^high^ monocytes by inhibiting monocyte migration from BM.

### miR-33 deficiency in hematopoietic stem cells expands myeloid progenitors by increasing *Hmga2* expression.

**FIG 5:**
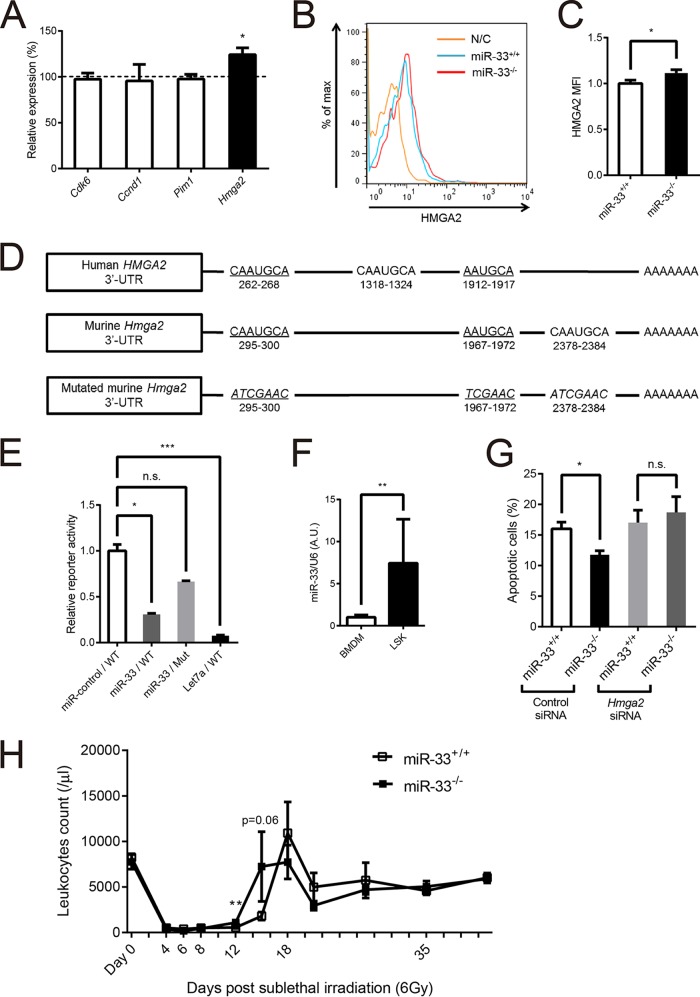
*Hmga2* is a target of miR-33 and reduced apoptosis of LSK cells. (A) Quantitative real-time PCR analysis of several genes that can affect a number of hematopoietic stem cells and be targets of miR-33 in LSK cells from miR-33^+/+^ and miR-33^−/−^ mice (miR-33^+/+^, *n* = 9; miR-33^−/−^ mice, *n* = 10). *, *P* < 0.05 (by Mann-Whitney *U* test). The dashed line indicates the level of each gene in miR-33^+/+^ mice. (B) Representative flow cytometric profiles of HMGA2 protein levels of LSK cells from miR-33^+/+^ and miR-33^−/−^ mice. N/C, unstained negative control. (C) HMGA2 protein levels in LSK cells from miR-33^+/+^ and miR-33^−/−^ mice (*n* = 10 per group). *, *P* < 0.05 (by Student's *t* test). (D) Diagram depicting the 3′ UTR of the human and murine *HMGA2* genes and the murine *Hmga2* 3′ UTR mutant, which was used for the 3′ UTR reporter assay. Underlining indicates miR-33 target sites conserved between human and mouse. Murine miR-33 target sites were replaced with scrambled sequences of the miR-33 seed sequence (italics). (E) 293T cells were transfected with WT or mutant *Hmga2* 3′ UTR luciferase constructs, along with expression plasmids for miR-control (negative control), miR-33, or let-7a (positive control) known to target *Hmga2* (*n* = 5 per group). *, *P* < 0.05; ***, *P* < 0.001; ns, not significant (by Kruskal-Wallis test). (F) Quantitative PCR analysis of miR-33 expression levels in BM-derived macrophages (BMDM) and LSK cells from WT mice (BMDM, *n* = 8; LSK, *n* = 5). **, *P* < 0.01 (by Mann-Whitney *U* test). (G) LSK cells collected from miR-33^+/+^ and miR-33^−/−^ mice using a cell sorter were transfected with control or *Hmga2* siRNA. Apoptosis of these cells was analyzed using annexin V (*n* = 5 per group). *, *P* < 0.05 (by Kruskal-Wallis test). (H) Leukocyte counts of miR-33^+/+^ and miR-33^−/−^ mice irradiated with 6 Gy of gamma rays at 8 weeks of age (miR-33^+/+^, *n* = 4 to 6 mice; miR-33^−/−^, *n* = 6 mice). *, *P* < 0.05 (by Mann-Whitney *U* test). All data are shown as means ± SEM.

We looked for the target genes of miR-33 which could be responsible for the hematopoietic phenotype of miR-33^−/−^ mice. Cyclin-dependent kinase 6 (CDK6) and cyclin D1 are well known as regulators of the cell cycle (21). Hematopoietic-specific human PIM1 transgenic mice show the expansion of hematopoietic stem cells (22). Overexpression of high-mobility group AT-hook 2 (HMGA2) in hematopoietic stem cells enhances their repopulation capacity and reduces their apoptosis in hematopoietic stem cells (23), and transgenic mice carrying a 3′ untranslated region (UTR)-truncated *Hmga2* show myeloid expansion (24). All of these genes are reported to be targets of miR-33 (25–27). We sorted LSK cells from miR-33^+/+^ and miR-33^−/−^ mice and analyzed the mRNA levels of these genes by quantitative PCR (qPCR). Only the *Hmga2* expression level was found to be significantly increased in miR-33-deficient LSK cells (Fig. 5A). The protein level of HMGA2 assessed by mean fluorescence intensity (MFI) using flow cytometry was also increased in miR-33-deficient LSK cells (Fig. 5B and C). Both human and murine HMGA2 proteins have three putative miR-33 binding sites in their 3′ UTRs, and two of them are conserved (Fig. 5D). Overexpression of miR-33 reduced the luciferase activity of a reporter gene fused with the murine *Hmga2* 3′ UTR in 293T cells. Mutation in these binding sites abolished the reduction of luciferase activity. Overexpression of let-7a was used as a positive control because there are seven binding sites for let-7a in the 3′ UTR of *Hmga2* (Fig. 5E) (28, 29). Additionally, miR-33 expression in LSK cells was higher than that in BM-derived macrophages (Fig. 5F). As mentioned above, HMGA2 in hematopoietic stem cells can reduce their apoptosis without proliferation and induce myeloid expansion (23, 24). These phenotypes are consistent with the hematopoietic phenotype of miR-33^−/−^ mice. To confirm that enhanced *Hmga2* expression in hematopoietic stem cells actually contributed to the reduction of their apoptosis in miR-33^−/−^ mice, we sorted LSK cells from miR-33^+/+^ and miR-33^−/−^ mice, transduced control and *Hmga2* small interfering RNAs (siRNAs) into them, and then assessed the percentage of apoptotic cells by flow cytometry using annexin V and PI at 48 h posttransduction. Results showed that the reduction of apoptosis in miR-33-deficient LSK cells was abolished with the transduction of *Hmga2* siRNA (Fig. 5G). On the other hand, the recovery from the nadir post-sublethal irradiation was earlier in miR-33^−/−^ mice than in miR-33^+/+^ mice (Fig. 5H). These results indicate that the expression of HMGA2 is increased in hematopoietic stem cells and that it contributes to the expansion of myeloid cells in miR-33-deficient BM by suppressing apoptosis in hematopoietic stem cells.

### Elevated HDL cholesterol in miR-33^−/−^ mice can suppress the emigration of Ly6C^high^ monocytes from BM.

**FIG 6:**
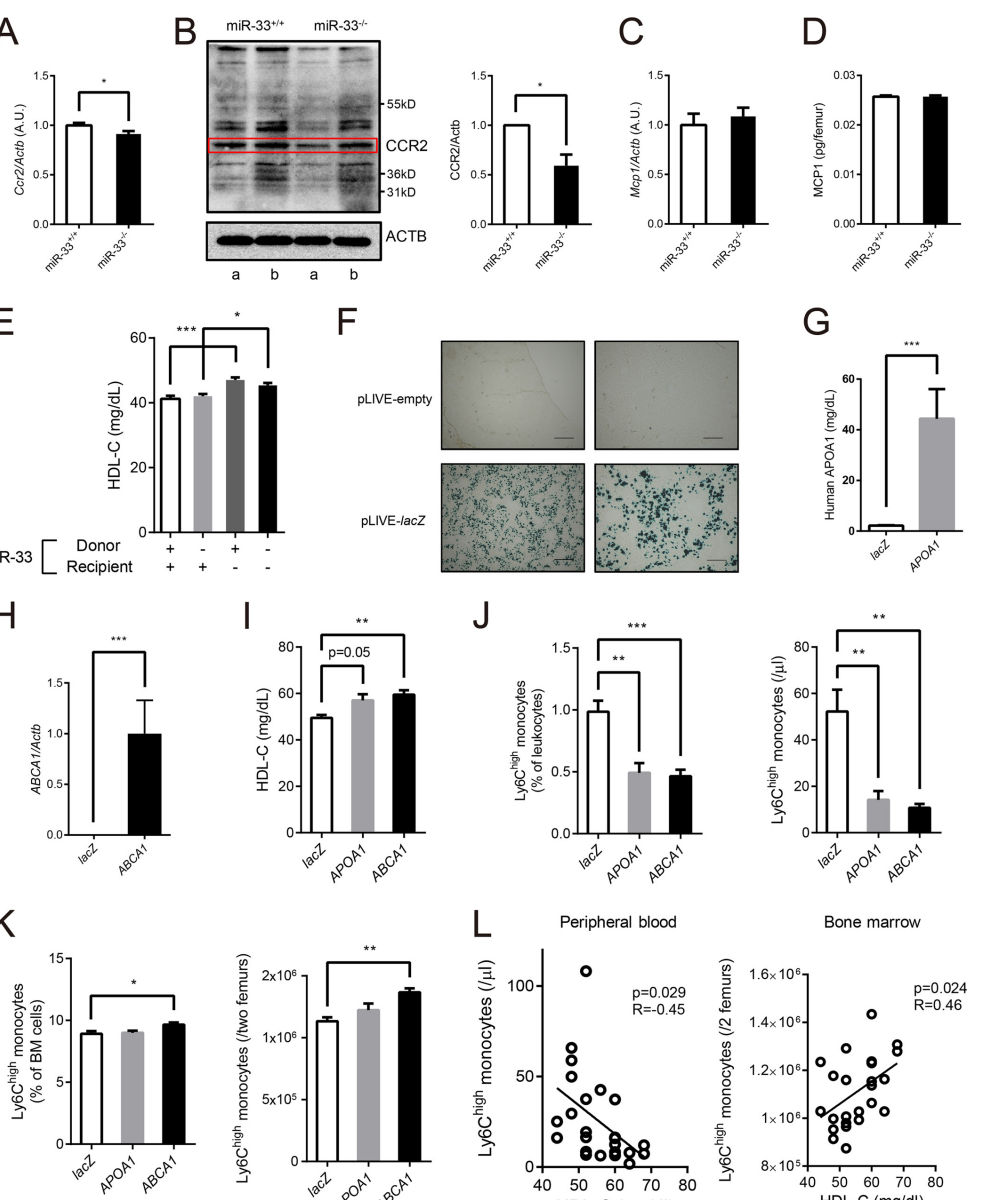
Hepatic ABCA1 increased HDL-C and repressed the egress of Ly6C^high^ monocytes from bone marrow. (A) Quantitative real-time PCR analysis of *Ccr2* in Ly6C^high^ monocytes in BM from miR-33^+/+^ and miR-33^−/−^ mice (*n* = 12 per group). *, *P* < 0.05 (by Student's *t* test). AU, arbitrary units. (B) Representative Western blotting of CCR2 in Ly6C^high^ monocytes in BM from miR-33^+/+^ and miR-33^−/−^ mice (left) and densitometry of three independent experiments (right). Matching letters indicate samples from the same experiment, and each experiment was performed with mixed protein obtained from 4 mice (*n* = 4 per group). β-Actin was used as a loading control. *, *P* < 0.05 (by Student's *t* test). (C) Quantitative real-time PCR analysis of *Mcp1* in whole BM cells obtained from miR-33^+/+^ and miR-33^−/−^ mice (*n* = 6 per group). (D) MCP1 protein levels in one femur from miR-33^+/+^ and miR-33^−/−^ mice assessed by ELISA (*n* = 6 per group). (E) Serum HDL-C levels in four groups of mice in the BMT experiment (*n* = 57 to 66 per group). *, *P* < 0.05; ***, *P* < 0.001 (by one-way ANOVA with a Turkey *post hoc* test). (F) β-Galactosidase staining of the liver of WT mice 2 days after injection of pLIVE-empty and pLIVE-*lacZ* vectors. Scale bars, 500 μm (left) and 200 μm (right). (G) Human APOA1 protein levels in PB from WT mice 3 weeks after injection of pLIVE-*lacZ* and pLIVE-*APOA1* vectors assessed by ELISA (*n* = 8 per group). ***, *P* < 0.001 (by Mann-Whitney *U* test). (H) Quantitative real-time PCR analysis of human *ABCA1* expression in the liver of WT mice at 3 weeks after injection of pLIVE-*lacZ* and pLIVE-*ABCA1* vectors (*n* = 8 per group). ***, *P* < 0.001 (by Mann-Whitney *U* test). (I) Serum HDL-C levels in WT mice 2 weeks after injection of pLIVE-*lacZ*, pLIVE-*APOA1*, or pLIVE-*ABCA1* (*n* = 8 per group). **, *P* < 0.01 (by Kruskal-Wallis test). (J) The proportion (left) and the number (right) of Ly6C^high^ monocytes in PB in WT mice 3 weeks after injection of pLIVE-*lacZ*, pLIVE-*APOA1*, or pLIVE-*ABCA1* (*n* = 8 per group). **, *P* < 0.01; ***, *P* < 0.001 (by Kruskal-Wallis test). (K) The proportion (left) and the number (right) of Ly6C^high^ monocytes in BM in WT mice 3 weeks after injection of pLIVE-*lacZ*, pLIVE-*APOA1*, or pLIVE-*ABCA1* (*n* = 8 per group). *, *P* < 0.05; **, *P* < 0.01 (by Kruskal-Wallis test). (L) Linear regression between serum HDL-C level and Ly6C^high^ monocyte counts in PB and BM (*n* = 24). All data are shown as means ± SEM.

**FIG 7:**
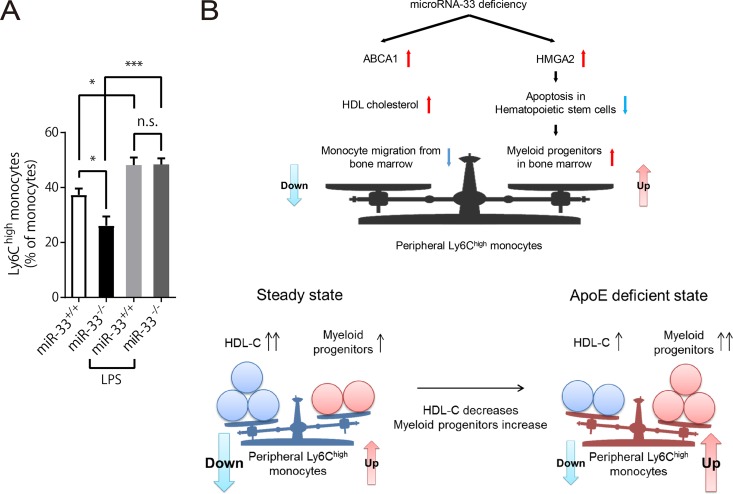
The balance between the hematopoietic and nonhematopoietic factors determines the population of peripheral Ly6C^high^ monocytes under miR-33-deficient conditions. (A) The proportion of Ly6C^high^ monocytes in peripheral monocytes from miR-33^+/+^ and miR-33^−/−^ mice at 36 h after PBS or LPS treatment (intraperitoneal injection of 5 μg/g of LPS) (*n* = 5 per group). *, *P* < 0.05; ***, *P* < 0.001; ns, not significant (by one-way ANOVA with Tukey *post hoc* test). (B) Diagram of the balance of factors which regulate the population of peripheral Ly6C^high^ monocytes under the miR-33-deficient condition.

C-C chemokine receptor type 2 (CCR2) and monocyte chemotactic protein-1 (MCP-1), also known as CCL2, are key regulators of Ly6C^high^ monocyte emigration from BM (30–33). MCP-1 secreted from the BM niche attracts Ly6C^high^ monocytes, which have CCR2 as a receptor, and induces egress of these monocytes from BM to peripheral circulation. Therefore, we assessed the expression of these two molecules. Both mRNA and protein expression of CCR2 were significantly reduced in BM Ly6C^high^ monocytes in miR-33^−/−^ mice. (Fig. 6A and B). On the other hand, MCP-1 expression in BM did not change (Fig. 6C and D). Thus, it was possible that the nonhematopoietic factors in miR-33^−/−^ mice reduced CCR2 expression in BM Ly6C^high^ monocytes. We hypothesized that this nonhematopoietic factor was increased in HDL-C in miR-33^−/−^ mice because it was reported that HDL-C suppresses the expression of CCR2 and migration of human monocytes (34, 35). Serum HDL-C was actually increased in miR-33-deficient recipient mice regardless of the phenotype of donor mice in the BMT study (Fig. 6E). Therefore, we thought that it was possible that increased HDL-C in miR-33^−/−^ mice inhibited the egress of Ly6C^high^ monocytes from BM by suppressing their CCR2 expression. To confirm this hypothesis, we transduced human *ABCA1* and *APOA1* into the liver of wild-type (WT) mice using a pLIVE (live *in vivo* expression) vector system to increase their HDL-C levels. These genes were transduced only into hepatocytes because they are driven by mouse alpha-fetoprotein enhancer II and minimal mouse albumin promoter. pLIVE vectors which expressed either human *ABCA1* or *APOA1* or *lacZ* as a control were delivered to the mouse liver by injection of the hydrodynamic tail vein. First, we transduced pLIVE-*lacZ* vectors into WT mice to confirm the efficacy of the system with β-galactosidase staining (Fig. 6F). Human APOA1 was actually secreted in serum at 3 weeks after transduction of pLIVE-*APOA1* (Fig. 6G), and expression of human ABCA1 was detected in the liver at 3 weeks after transduction of pLIVE-*ABCA1* (Fig. 6H). Serum HDL-C was increased in mice transduced with both pLIVE-*APOA1* and pLIVE-*ABCA1* (Fig. 6I), and peripheral Ly6C^high^ monocytes were decreased in these mice (Fig. 6J). Meanwhile, Ly6C^high^ monocytes in BM tended to increase (Fig. 6K). Moreover, linear regression analyses using the complete data set of this experiment showed that the serum HDL-C level is negatively correlated with the peripheral Ly6C^high^ monocyte count and positively correlated with the number of Ly6C^high^ monocytes in BM (Fig. 6L). These results indicate that the increase in HDL-C can suppress the emigration of Ly6C^high^ monocytes from the BM to PB, which is compatible with our hypothesis and mimics the nonhematopoietic phenotype of miR-33^−/−^ mice. To further confirm the suppressed emigration of Ly6C^high^ monocytes from BM in miR-33^−/−^ mice, we forced monocytes to emigrate from BM by lipopolysaccharide (LPS) stimulation. The difference in proportions of Ly6C^high^ monocytes in peripheral monocytes between miR-33^+/+^ and miR-33^−/−^ mice was abolished at 36 h post-LPS treatment (Fig. 7A).

## DISCUSSION

In the current study, we demonstrated the novel mechanism that miR-33 regulates the population of peripheral Ly6C^high^ monocytes through at least two different pathways. One pathway involves enhanced expression of *Hmga2* in hematopoietic stem cells. *Hmga2* is reported to be a potential target of miR-33 and to regulate self-renewal and apoptosis of cells (23, 24, 27). In the BMT study, mice transplanted with miR-33^−/−^ BM showed an increase in myeloid progenitors and Ly6C^high^ monocytes in BM and a decrease in apoptosis in LSK cells in which *Hmga2* expression was increased. These phenotypes are consistent with previous reports that transgenic mice carrying a 3′ UTR-truncated *Hmga2* showed myeloid expansion (24) and overexpression of *Hmga2* in hematopoietic stem cells before BMT enhanced hematopoietic stem cell repopulation capacity and reduced their apoptosis (23). Enhanced expression of *Hmga2* in miR-33-deficient hematopoietic stem cells itself can increase peripheral Ly6C^high^ monocytes because the proportion of peripheral Ly6C^high^ monocytes derived from miR-33^−/−^ BM was higher than that derived from miR-33^+/+^ BM in the same recipient in the competitive repopulation study. It was also demonstrated that miR-33^−/−^ mice recovered earlier from the nadir post-sublethal irradiation than miR-33^+/+^ mice, which could be due to the reduction of apoptosis in hematopoietic stem cells as a result of enhanced *Hmga2* expression. Therefore, anti-miR-33 oligonucleotide treatment may help patients to recover from the nadir post-cancer treatment.

The second pathway involves an increase in HDL-C level. Ly6C^high^ monocytes in PB were decreased in miR-33^−/−^ mice compared with the level in miR-33^+/+^ mice. Meanwhile, Ly6C^high^ monocytes in the BM were increased. This indicates that miR-33 deficiency suppressed the emigration of Ly6C^high^ monocytes from BM to PB. Emigration of monocytes from BM is mainly mediated by CCR2 in monocytes and by MCP-1 secreted from BM mesenchymal stem cells and their progeny (30–33). Moreover, it was also reported that HDL-C reduced CCR2 expression in human monocytes (34, 35). In a previous study, we demonstrated that serum HDL-C in miR-33^−/−^ mice increased by up to 40% (7). An increase in serum HDL-C was also observed in miR-33-deficient recipient mice regardless of the expression of miR-33 in donor mice in the BMT study, and these mice showed decreased peripheral Ly6C^high^ monocytes. Furthermore, the expression of *Ccr2* in BM Ly6C^high^ monocytes was decreased in miR-33^−/−^ mice. Therefore, we speculated that the increase in the HDL-C level in miR-33^−/−^ mice caused the suppression of monocyte emigration from BM. To confirm this hypothesis, we transduced human ABCA1 or APOA1 into the liver of WT mice to increase HDL-C using pLIVE vectors. Peripheral Ly6C^high^ monocytes in these mice were decreased. On the other hand, BM Ly6C^high^ monocytes were increased. These results indicate that an increase in HDL-C can suppress the emigration of Ly6C^high^ monocytes from the BM to PB.

In our previous study, the proportion of peripheral Ly6C^high^ monocytes in miR-33^−/−^ *Apoe*^−/−^ mice was increased compared to that in *Apoe*^−/−^ mice (8). Thus, we were surprised at the reduction of peripheral Ly6C^high^ monocytes in miR-33^−/−^ mice compared to that in miR-33^+/+^ mice under ApoE-sufficient conditions. According to the results of the present study, we have the following hypothesis about the mechanism of this discrepancy. As described above, miR-33 deficiency in hematopoietic stem cells increases peripheral Ly6C^high^ monocytes. Meanwhile, miR-33 deficiency in nonhematopoietic cells increases HDL-C by enhancement of ABCA1 expression, which suppresses the egress of Ly6C^high^ monocytes from BM to PB. We hypothesized that the balance between these two components determines the number and proportion of peripheral Ly6C^high^ monocytes (Fig. 7B). In fact, forced induction of monocyte egress from BM by LPS abolished the reduction of peripheral Ly6C^high^ monocytes in miR-33^−/−^ mice (Fig. 7A). It is known that ApoE deficiency causes the proliferation and expansion of hematopoietic stem cells and a marked reduction in HDL-C level (36). Therefore, the hematopoietic phenotype observed in miR-33^−/−^ mice can be enhanced, and the nonhematopoietic phenotype can be diminished on ApoE-deficient background. Consequently, peripheral Ly6C^high^ monocytes can increase in miR-33^−/−^ *Apoe*^−/−^ mice.

We along with other groups have reported that miR-33 targets ABCA1, and its deficiency or inhibition by anti-miR oligonucleotides increased HDL-C and enhanced macrophage cholesterol efflux (4–7). However, it is still not clear whether inhibition of miR-33 has a favorable effect on atherosclerosis (8, 16–19). Moreover, it is also still unclear which contributes more to antiatherosclerosis effects, increased HDL-C or enhanced macrophage efflux, with miR-33 inhibition. We think that the balance of hematopoietic and nonhematopoietic factors described above may explain this inconsistency. Ly6C^high^ monocytes are involved in inflammation and thought to be inflammatory monocytes in mice. These monocytes are the main monocyte subset recruited into atherosclerotic plaques, and some of them differentiate into foamy macrophages (10, 37). Moreover, reduction of Ly6C^high^ monocytes induced by depletion of CD8^+^ T cells attenuates atherosclerosis progression (38). Therefore, we think that hematopoietic miR-33 deficiency, which increases peripheral Ly6C^high^ monocytes, is proatherogenic and that nonhematopoietic miR-33 deficiency, which reduces peripheral Ly6C^high^ monocytes, is atheroprotective, and the balance of these two factors affects atherosclerosis formation. For example, even in *Ldlr*^−/−^ mice, the increase in HDL-C by inhibition of miR-33 may be mild or modest relative to the level in WT mice (16, 17) and thus may diminish the suppressive effect of miR-33 on monocyte migration from BM; *Ldlr*^−/−^ mice are also known to show myeloid expansion and monocytosis on a high-fat diet although the effects are less severe than in *Apoe*^−/−^ mice (36, 39), which can increase the effect of miR-33 on myeloid progenitors. Consequently, the balance described above might be tilted toward increasing peripheral inflammatory Ly6C^high^ monocytes in *Ldlr*^−/−^ mice as it is in *Apoe*^−/−^ mice (Fig. 7B), which might diminish the anti-atherosclerotic effect of miR-33 inhibition. Moreover, a recent report suggested that Ly6C^high^ monocytes are also important to plaque regression (40). Thus, the effects of miR-33 may be different even between plaque progression and regression. In addition, there are many factors that can affect this balance, such as circumstances of the animal facility, the type of diet and duration, and so on. Thus, all of these factors should be taken into account to determine the effect of miR-33 on atherosclerosis.

One microRNA has more than 100 target genes (41) and can understandably regulate several genes which affect the same phenotype. However, this has not yet been directly demonstrated. In the present study, we showed that miR-33 regulates the population of peripheral Ly6C^high^ monocytes by targeting two different genes which function oppositely.

It is possible that some other miR-33 target genes also affect the phenotypes observed in the current study. Moreover, we did not directly demonstrate that the balance of hematopoietic and nonhematopoietic factors under the miR-33-deficient condition mentioned above affects atherosclerotic plaque formation. Thus, further exploration of miR-33 function is necessary to understand its precise effect on atherosclerosis.

In conclusion, miR-33 deficiency influences the population of Ly6C^high^ monocytes through *Hmga2*-dependent hematopoietic and HDL-C-dependent nonhematopoietic pathways. The balance of these two components may affect inflammatory diseases, including atherosclerotic plaque formation. Cell-type-specific or organ-specific miR-33 inhibition would be necessary for more effective application as atherosclerotic therapy.

## MATERIALS AND METHODS

### Mice.

miR-33-deficient mice were generated as described previously (7). Ly5.1 (CD45.1^+^) mice were provided by Shigekazu Nagata. Both types of mice were generated on the C57BL/6 background and maintained in our pathogen-free environment. All experiments except for BMT experiments were performed using male mice. All of the experimental protocols were approved by the Ethics Committee for Animal Experiments of Kyoto University.

### Complete blood cell count.

PB was obtained from the inferior vena cava of anesthetized mice and put in EDTA-coated tubes. Complete blood cell counts were quantified from whole blood using a hematology cell counter (Celltac α MEK-6358; Nihon Kohden).

### Flow cytometry.

In analyzing the population of peripheral leukocytes, PB was collected from the inferior vena cava of anesthetized mice. Samples were blocked with anti-CD16/CD32 antibody (Fc block; BD Pharmingen) for 15 min at 4°C and then labeled with antibodies for 30 min at room temperature. Red blood cells (RBCs) were lysed using a commercial RBC lysis solution (BD PharmLyse; BD Biosciences). To analyze the population of BM cells, they were collected by flushing femurs with phosphate-buffered saline (PBS) supplemented with 2% fetal bovine serum (FBS). The suspension was passed through a 40-μm-pore-size nylon mesh (Cell Strainer; BD Bioscience). After red blood cells were lysed, BM cells were stained with antibodies for 30 min at 4°C. Doublets were excluded, and live cells were identified as PI-negative cells. Antibodies were purchased as follows: CD115 (AFS98), Ly6C (HK1.4), CD11b (M1/70), CD16/32 (93), CD34 (RAM34), and IL7a (A7R34) were from eBioscience; CD3 (17A2), CD45R/B220 (RA3-6B2), Ly6G (1A8), c-Kit (2B8), Sca-1 (D7), CD45.1 (A20), and CD45.2 (104) were from BioLegend; a lineage antibody cocktail consisting of CD3 (145-2C11), CD11b (M1/70), CD45R/B220 (RA3-6B2), TER-119 (Ly76), and Gr-1 (RB6-8C5) was from BD Pharmingen. The total BM cells in two femurs were counted using a Z1 particle counter (Beckman Coulter). For apoptosis analysis, samples were labeled with annexin V-Alexa Fluor 488 (Thermo Fisher Scientific) after antibodies were washed off. For intracellular staining, after cell surface molecules were stained, antibodies were washed, and samples were fixed and permeabilized with Cytofix/Cytoperm buffer and Cytoperm Plus buffer (BD Pharmingen) and then stained with anti-Ki-67–fluorescein isothiocyanate (FITC) antibody (clone 16A8; BioLegend) and DAPI for cell cycle analysis. For analysis of the protein level of HMGA2 in LSK cells, after fixation and permeabilization samples were incubated with an anti-HMGA2 antibody (clone D1A7; Cell Signaling). Antibodies were washed off, and then samples were labeled with anti-rabbit IgG–Alexa Fluor 488 antibody (clone Poly4064; BioLegend). Data were acquired using a BD FACSAria II flow cytometer and analyzed with BD FACSDiva software (BD Biosciences).

### Colony formation assay.

BM cells (2.0 × 10^4^ cells) were cultured in methylcellulose-based medium containing a cocktail of recombinant cytokines including stem cell factor (SCF), interleukin-3 (IL-3), IL-6, and Epo (MethoCult; Stemcell Technologies). The colonies in each dish were counted after 10 days of incubation.

### Bone marrow transplantation.

Male mice with genotypes of miR-33^+/+^ and miR-33^−/−^ were used as donors. Recipients were female miR-33^+/+^ and miR-33^−/−^ mice. Both donor and recipient mice were 8 weeks old. Donors were euthanized by cervical dislocation, and BM cells were collected by flushing femurs and tibias as mentioned above. Red blood cells were lysed using ammonium chloride-potassium (ACK) lysing buffer (Lonza). BM cells were then washed twice with PBS supplemented with 2% FBS. To induce BM aplasia, recipients were irradiated with two doses of 6 Gy at an interval of 3 h (cesium 137; Gammacell 40 Exactor) and injected intravenously with 2.0 × 10^6^ BM cells 6 h after irradiation. When mice were 22 weeks old (i.e., 14 weeks after BMT), the populations of Ly6C^high^ monocytes in PB and in the BM and myeloid progenitors in BM were analyzed by flow cytometry. The hematologic chimerism of mice was determined by PCR using genomic DNA from the BM and tail and measuring miR-33 levels in BM cells by quantitative PCR at 22 weeks.

### Competitive repopulation assay.

Ly5.1 mice were lethally irradiated as described above, and then their BM was reconstituted with mixtures of BM cells from CD45.1^+^ miR-33^+/+^ mice and CD45.2^+^miR-33^+/+^ or miR-33^−/−^ mice at a ratio of 1:1. The proportion of CD45.2^+^ cells in PB was analyzed every 2 weeks from 8 to 14 weeks post-BMT. Finally, the proportion of CD45.2^+^ hematopoietic progenitor cells in the BM was assessed at 14 weeks post-BMT.

### RNA extraction and qRT-PCR.

Total RNA was isolated and purified using TRI reagent (Sigma-Aldrich) or NucleoSpin RNA XS (Macherey-Nagel), and cDNA was synthesized using a Verso cDNA synthesis kit (Thermo Fisher Scientific) or SuperScript VILO cDNA synthesis kit (Thermo Fisher Scientific) in accordance with the manufacturer's instructions. For quantitative real-time PCR (qRT-PCR), specific genes were amplified using 40 cycles with Thunderbird SYBR qPCR mix (Toyobo). Expression was normalized to the housekeeping gene *Actb*. Gene-specific primers are summarized as follows: Hmga2 sense, AAGGCAGCAAAAACAAGAGC; Hmga2 antisense, AATCCTCCTCTGCGGACTCT; Cdk6 sense, TGTTTCAGCTTCTCCGAGGT; Cdk6 antisense, CTGGACTGGAGCAGGACTTC; Pim1 sense, AGATCATCAAGGGCCAAGTG; Pim1 antisense, GGATGGTTCCGGATTTCTTC; Ccr2 sense, ATCTGCTCAACTTGGCCATC; Ccr2 antisense, CCCAAAGACCCACTCATTTG; Mcp1 sense, CTGGATCGGAACCAAATGAG; Mcp1 antisense, TGAGGTGGTTGTGGAAAAGG; human *ABCA1* sense, CCAAGAAGTTTCTGAGCTTTGTGGCC; human *ABCA1* antisense, AGTCCCAAGACTATGCAGCAATGTTTTTG; *Actb* sense, GATCTGGCACCACACCTTCT; *Actb* antisense, GGGGTGTTGAAGGTCTCAAA.

### Quantitative PCR for microRNAs.

miR-33 was measured in accordance with a TaqMan microRNA assay (Applied Biosystems) protocol, and the products were analyzed using a thermal cycler (StepOnePlus; Applied Biosystems). Samples were normalized by U6 snRNA expression.

### Western blotting.

Western blotting was performed using standard procedures as described previously (42). A total of 4 μg of protein was fractionated using NuPAGE 4% to 12% Bis-Tris gels (Invitrogen) and transferred to a Protran nitrocellulose transfer membrane (Whatman). The membrane was blocked using PBS containing 5% nonfat milk for 30 min and incubated with the primary antibody (anti-CCR2, 1:1,000; anti-β-actin, 1:3,000) overnight at 4°C. Following a washing step in PBS–0.05% Tween 20 (0.05% T-PBS), the membrane was incubated with the secondary antibody (anti-rabbit or anti-mouse horseradish peroxidase [HRP]-linked IgG; 1:2,000) for 1 h at 4°C. The membrane was then washed in 0.05% T-PBS and detected using Pierce Western blotting substrate or Plus substrate (Thermo Fisher Scientific) and an ImageQuant LAS 4000 Mini imager (GE Healthcare).

### Quantification of MCP1 in BM and serum human APOA1 levels.

For quantification of MCP1 levels in the BM, each femur was ground in a mortar with 300 μl of tissue extraction buffer (150 mM NaCl, 1 mM EGTA, 1 mM EDTA, 1% Triton X-100, 0.5% sodium deoxycholate, and protease inhibitor cocktail in 100 mM Tris buffer, pH 7.4) using a pestle. Samples were collected in a 1.5-ml tube, placed on an orbital shaker for 2 h at 4°C, and then centrifuged for 20 min at 13,000 rpm at 4°C. Supernatants were used for the assay. MCP1 levels were quantified using a mouse/rat CCL2/JE/MCP-1 Quantikine enzyme-linked immunosorbent assay (ELISA) kit (R&D Systems) in accordance with the manufacturer's instructions. Serum human APOA1 levels were measured using a human apolipoprotein A-I Quantikine ELISA kit (R&D Systems).

### Dual-Luciferase assay.

A Renilla luciferase reporter plasmid with the 3′ UTR of mouse *Hmga2* was purchased from Addgene. To create reporter plasmids with mutant 3′ UTR sequences, a QuikChange II XL site-directed mutagenesis kit (Agilent Technologies) was used. In brief, 293T cells were cotransfected with reporter plasmids with a WT or mutant 3′ UTR of *Hmga2* and expression plasmids for an miR control (negative control), let-7a (positive control), or miR-33. As an internal control reporter, a reporter vector with firefly luciferase was also cotransfected to normalize the transduction efficiency. Luciferase activities were measured using a Dual-Luciferase kit (PicaGene dual kit; Toyo Ink Co.). The relative luciferase activity of each construct (arbitrary units) was reported as the fold induction.

### Lentiviral vectors and transduction.

For knockdown of *Hmga2*, a pGIPZ system (GE Dharmacon) was utilized. We validated the efficiencies of six clones of *Hmga2* short hairpin RNA (shRNAs) using HB2 cells, which were provided by Yuko Okamatsu, and used the most efficient one (target site, GAGATAACTAGCTGAGTAA) for the experiment. We also confirmed that the clone was efficient even when transfected into LSK cells. Lentiviral particles were produced utilizing 293T cells and concentrated by ultracentrifugation (25,000 rpm for 2 h). Sorted LSK cells were cultured with control or shRNA virus in the presence of 8 μg/ml Polybrene, 10 ng/ml murine IL-3 (mIL-3), 10 ng/ml mIL-6, 50 ng/ml SCF, and 100 μg/ml Primocin. The medium was replaced 24 h after transduction and analyzed 48 h after transduction.

### pLIVE vectors and transduction.

To overexpress human *APOA1* and *ABCA1* in the liver of WT C57BL/6 mice, we utilized pLIVE *in vivo* expression and reporter vectors (Mirus). Full-length coding regions of *APOA1* and *ABCA1* were amplified from human cDNA and inserted into the multicloning site of a pLIVE vector. These vectors (50 μg/mouse) were injected with TransIT-EE delivery solution (Mirus) into the tail vein of WT mice in accordance with the manufacturer's instructions.

### Statistics.

Data are presented as means ± standard errors of the means (SEM). Statistical comparisons were performed using nonparametric analysis when the group numbers were less than 10 (Mann-Whitney *U* test). Otherwise, an unpaired two-tailed Student *t* test was conducted. In dealing with experiments that included more than two groups, statistical significance was tested by a Kruskal-Wallis test when the group numbers were less than 10. Otherwise, one-way analysis of variance with the Tukey's *post hoc* test was conducted. The level of significance was set at a probability value of less than 0.05.
